# Tempo and Mode in the Molecular Evolution of Influenza C

**DOI:** 10.1371/currents.RRN1199

**Published:** 2010-12-07

**Authors:** Derek Gatherer

**Affiliations:** MRC-University of Glasgow Centre for Virus Research, 8 Church Street, Glasgow G11 5JR, UK.

## Abstract

Abstract:

Influenza C contributes to economic damage caused by working days lost through absence or inefficiency and may occasionally cause an acute respiratory illness in a paediatric setting. All Influenza C sequences from the NCBI Influenza Virus Resource were examined to determine the date of the most recent common ancestor (t-MRCA), the average nucleotide substitution rate, and the location of residues under positive selection, for each of the seven genome segments of this virus. The segment with the deepest phylogeny was found to be segment 4, encoding the haemagglutinin-esterase protein (HE) with mean t-MRCA at 1890 of the common era (AD), at a 95% highest posterior density (HPD) of 1857-1924 AD. Other genome segments have slightly more recent common ancestors, ranging from mean t-MRCAs of 1916 AD (HPD 1891-1937) for segment 7, encoding the two non-structural proteins (NS) to 1944 AD (HPD 1940-1948) for segment 2 encoding the type 1 basic polymerase (PB1). On the basis of the Bayesian analysis a reclassification of lineages within genome segments is proposed. Some evidence for positive selection was found in the receptor-binding domain of the haemagglutinin-esterase protein. However, average ω (omega) values ranged from 0.05 for polymerase basic protein 2 (PB2) to 0.38 for non-structural protein 2 (NS2), suggesting that strong to moderate purifying selection is the main trend. Characteristic combinations of segment lineages were identified (genome constellations) and shown to have a relatively short life-span before being broken up by reassortment.

## Introduction

Influenza C virus (genus Influenzavirus C; family Orthomyxoviridae) causes a sore throat, coryza, headache and malaise.  A fever is present in a third of cases [1], rising to 90% in children [2].  Symptoms can have a duration of up to 11 days, with an average incubation period of 4 days [1].  Although not constituting a pandemic threat, and not included in the seasonal influenza vaccine, Influenza C can cause acute respiratory illness in a paediatric setting [3] [4] [5], especially in those less than 2 years old [2].  In some outbreaks vomiting, diarrhoea and a high rate of hospitalization have been reported [6].  Influenza C also contributes to economic damage caused by working days lost through absence and decreased efficiency of the workforce during its relatively long period of presentation of symptoms [7]. Nearly half of inoculated volunteers develop symptoms, despite a high level of population seropositivity [1].  This suggests that Influenza C, like the other influenza viruses, is able to evade the host immune system via antigenic variation.  Knowledge of its patterns of molecular evolution is thus of importance to any future attempt to contain the disease or vaccinate against it. 

Like the other influenza viruses, Influenza C has a segmented RNA negative-stranded genome, but comprising 7 segments rather than the 8 found in Influenza B and Influenza A (reviewed in [8]).  This is due to the absence of a neuraminidase gene-encoding segment in Influenza C.  Also, the haemagglutinin gene found in the other two viruses is replaced by a haemagglutinin-esterase (HE) gene.  One further difference with the other viruses is that the matrix proteins (M1 and CM2) found on genome segment 6 (corresponding to segment 7 in Influenza A and B) are in frame [9].  Like the other viruses, the non-structural proteins (NS1 and NS2) found on segment 7 (corresponding to segment 8) are out of frame.  Typically of all influenza viruses, Influenza C exhibits reassortment of genome segments as a result of double infection of a host with two virus strains [3] [10] [11] [12].  HE is the major surface protein of Influenza C along with some CM2.  M1 is found under the envelope lipid bilayer along with a small amount of NS2.  The remaining gene products - basic polymerase proteins (PB) 1 and 2, a third polymerase protein (P3) and the nucleoprotein (NP) - are complexed with the viral RNA in a ribonucleoprotein.  NS1 is not found in the virion [8].    

Influenza C appears to be primarily a disease of humans, although occasionally it has been found in pigs and dogs [13] [14] [15].  Owing to surveillance campaigns initiated in the late 1980s, most of the extant sequences in the NCBI Influenza Virus Resource [16] are from Japan or other parts of East Asia.  The average rate of nucleotide substitution in HE has been calculated at 4.9 x 10^-4^ substitutions/site/year [17], an order of magnitude slower than the corresponding rate for the haemagglutinin gene of Influenza A.  Similar findings have been made for the NS genes [18] [19]. 

This paper presents the first comprehensive analysis of the molecular phylogeny of the complete available set of sequences of all seven segments of influenza C using Bayesian methods implemented in BEAST [20] [21] and the first analysis of positive selection.    

## Methods 

Sequences of the seven RNA genome segments of influenza C were downloaded from the Influenza Virus Resource (http://www.ncbi.nlm.nih.gov/genomes/FLU/ [16]).  For phylogenetic tree building and estimation of the date of the most recent common ancestor (t-MRCA), genome segment nucleotide sequences (rendered as DNA) were used.  For estimation of positive selection, cDNA sequences were used.  In the case of genome segment 7, the NS1 and NS2 genes are out of frame, and these cDNAs were analysed separately.  In genome segment 6, by contrast, the M1 and CM2 genes are in frame and were treated as a single cDNA.  In all seven genome fragments and all eight cDNAs, duplicate sequences were removed, retaining the oldest by date of isolation.  Alignment using Muscle [22] and best substitution model estimation were performed in MEGA [23] (http://www.megasoftware.net).  It is assumed that no homologous recombination occurs in Influenza C [24].  

Bayesian methods for tree building and substitution rate estimation were implemented in BEAST [20] [21] (http://beast.bio.ed.ac.uk).   Bayesian analysis enables the determination of the date of the most recent common ancestor (t-MRCA) of statistically well-supported clades under a relaxed evolutionary clock model.  10^8^ iterations were run in BEAST, sampling trees every 5000 iterations.  For all segments, the tree prior was set to a constant size coalescent and the molecular clock model to a relaxed uncorrelated lognormal, both of which were determined by Bayes Factor analysis to be the best choices.  Each genome segment alignment was analysed 10 times and their traces combined for the final parameter estimation in Tracer (http://beast.bio.ed.ac.uk/Tracer), using a burn-in of 10^7 ^ iterations for each replicate.  Convergence was achieved for all data sets.  Accurate estimation of substitution rates was assisted by the use of the maximum available time depth for sampling of strains, either from years 1950 to 2000 (segments 1,2,3 and 5), 1947 to 2000 (segments 6 and 7) or 1947 to 2009 (segment 4).  Maximum clade credibility (MCC) trees were constructed using the same burn-in with the TreeAnnotator function of BEAST and viewed in FigTree (http://beast.bio.ed.ac.uk/FigTree).  Within the phylogenetic tree of each genome segment, *lineages* were defined as clades of three or more members with a posterior probability of *p*>0.99, except in the case of genome segment 7 where *p*>0.96 was used.  Lineages are named according to the earliest strain within them.   

Positive selection was analysed on the cDNA sequence alignments using sitewise likelihood ratio [25] (http://www.ebi.ac.uk/goldman/SLR) and PAML [26] (http://abacus.gene.ucl.ac.uk/software/paml.html), to identify individual residue positions likely to be under positive selection and also overall ratios of non-synonymous to synonymous substitution.  Since prior cultivation of influenza strains in embryonated eggs can lead to adaptation and consequent artefactual identification of sites under positive selection [27], those sequences annotated in the Influenza Virus Resource or in the literature [11] [28] [29] [30] [31] [32] [33] as egg-cultured were excluded from the positive selection scan.   

Protein structures were viewed in MOE (http://www.chemcomp.com).    

## Results 

### Genome Segment Lineages 

Table 1 summarizes the division of the sequences of each segment into lineages.  Of the seven genome segments, six demonstrate clear lineages with fewer than ten strains that cannot be assigned to a lineage (referred to as outlier strains).  All lineages are defined as clades with posterior probability of *p*>0.99.  Segment 7, encoding the non-structural proteins, has one of its four lineages that is less well-supported (*p*>0.96, see Table 2) and a larger number of outlier strains. In most segments, the outlier strains are older, i.e. pre-1985, but in segment 1 and segment 3 there are outliers from the 1990s.  

Table 2 describes the size of each of the lineages, the dates of their most recent common ancestors (t-MRCAs) with corresponding 95% highest posterior densities (HPDs) and previous names according to Peng et al [3], Tada et al [12], Alamgir et al [10], Matsuzaki et al [4] and Muraki & Hongo [8].  All the lineages detected here by Bayesian phylogenetic analysis have been detected at least once in the literature using older neighbour-joining or parsimony methods, although previously their statistical support was less robust.  Some lineages found in the previous literature are not supported by the current analysis, for instance lineage III of segment 6 [12] and lineage Taylor/47 of segment 4 [4].  Others are combined into larger clades, for instance the SP82 and AI81 lineages of segment 4 [4] are now part of the C/Johannesburg/66 lineage and the M and A lineages of segment 6 [4] are now part of the C/Yamagata/64 lineage.  The present study therefore simplifies the lineage structure of the Influenza C genome segments and gives them a firmer Bayesian statistical grounding than was possible using previous neighbour joining or parsimony bootstrapping methods. 

**Figure:**
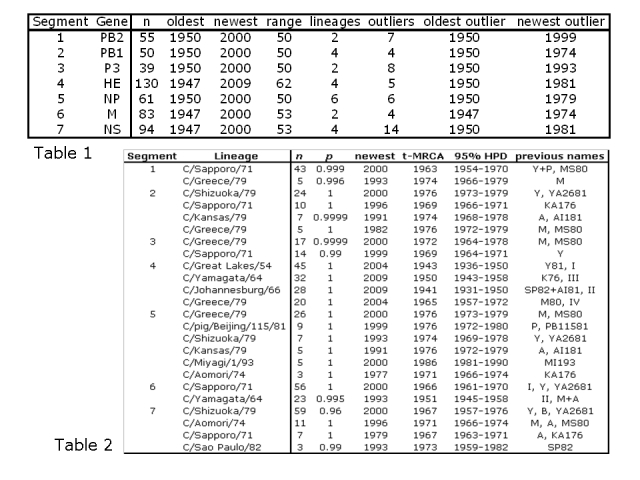
Table 1: Age distribution of sequences, number of lineages and age distribution of outliers (sequences not falling within lineages).  *n*: number of sequences.

Table 2:  Lineages identified by Bayesian analysis. *n*: number of strains in lineage; *p*: Bayesian posterior probability of monophyletic clade; newest: the date of the most recent strain identified in each lineage; t-MRCA: the mean date (in years of the Common Era, i.e. AD) of the most recent common ancestor of each lineage; 95% HPD: the 95% highest posterior densities of the date of the t-MRCA; previous name: the previous designations given to the lineages in the literature [3] [4] [8] [10] [12].

The oldest of the 24 lineages identified across the seven genome segments is the C/Johannesburg/66 lineage of fragment 4 with a t-MRCA date of 1941 (95% HPD 1931-1950) and the youngest is C/Miyagi/93 lineage of fragment 5 with a t-MRCA date of 1986 (95% HPD 1981-1990).  In general, fragment 4 has the oldest lineages (mean t-MRCA at 1950) and fragment 5 the youngest (mean t-MRCA at 1977).  The correlation coefficient between size of a lineage and its age is of only moderate intensity at 0.53, but is nevertheless significant as assessed by t-test at *p*<0.0001.  This suggests that many of the lineages may be gradually diversifying with age.  However, some of the lineages have fewer than ten members, so accurate estimation of their t-MRCA is difficult.   

Figure 1 shows the maximum clade credibility (MCC) phylogenetic tree for genome segment 5, encoding the nucleoprotein (NP).  The outliers are here defined as those strains that cannot be fitted into any lineage with posterior probability of greater than 0.99, and there are many nodes that can be seen to have low values.  A less conservative approach might admit C/California/78 to form a clade encompassing the C/Miyagi/93 clade and two other outliers at posterior probability of * p*=0.91, thus reducing the number of outliers to 3.  In general, this kind of relaxation has not been necessary except in the case of segment 7, where there are more outliers (see Table 1) and rigorous definition of clades is generally more difficult than in the other segments.  Even so, in segment 7 relaxation of the threshold was only necessary to *p*>0.96, compared with the* p*>0.99 used in all other segments.  

**Figure:**
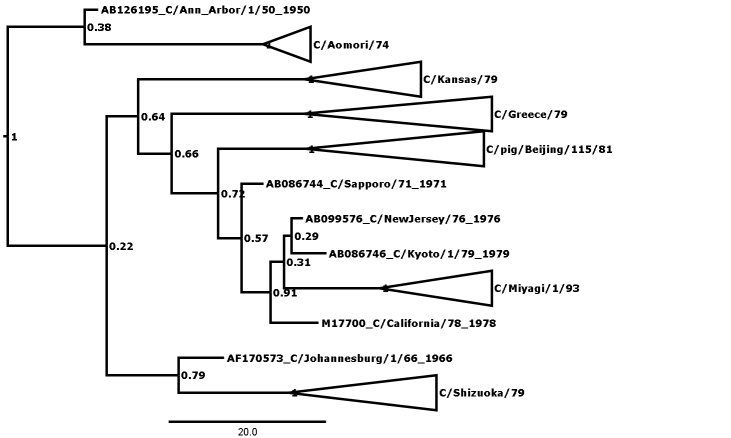
Figure 1:  MCC phylogenetic tree for segment 5, encoding the nucleoprotein (NP).  Bayesian posterior confidence values for the clades are given at each node.  Lineages (see Table 2) are collapsed into triangles.  The 6 outlier strains (see Table 1) are named in full.  The scale is in years.

The MCC phylogenetic trees for the other six genome segments are included as Supplementary Figures 1-6.  The FigTree input files are available on request from the author.    

### Genome Constellations 

24 strains from the period 1990-2000 in which all seven genome segments were available were allocated to their relevant lineage, as defined in Table 2, for each segment.  A total of nine different combinatorial configurations of lineages were found in the 24 strains.  These are designated as *genome constellations* (following the usage for Influenza B [34]) and are shown in Table 3.  

Constellation 1 consists of a group of strains dating from 1996 to 2000, and is divisible into two sub-groups, a and b, depending on the presence of either the C/Yamagata/64 or C/Great Lakes/54 lineages in fragment 4 (HE).  Constellation 2 is found in two strains from 1992-1993 and differs from both 1a and 1b in having C/Greece/79 as the fragment 4 (HE) lineage and C/Yamagata/64 as the fragment 6 (M) lineage.  Constellation 4 is divisible into two sub-groups, a and b, depending on the presence in segment 5 of the C/Miyagi/1/93 and C/pig/115/81 lineages respectively.  Constellations 1a, 1b, 2, 3 and 7 were previously identified as the RA176-related, YA2681-related, MS80-related, AI81-related and SP82-related “lineages” (note different use of the term lineage in this context by some previous authors) respectively [4].  However, constellations 4a, 4b, 5, 6 and 8 are described for the first time here.  

A selection of reference strains dating from 1947 to 1982 were similarly examined (Table 4).  It is notable that very few correspond to the constellations visible in the sample of strains from 1990 to 2000.   Only C/pig/115/Beijing/81 is identical to genome constellation 6 (found in C/Miyagi/3/91) and C/Sao Paulo/82 is identical to genome constellation 7 (found in C/Yamagata/1993).  Lineages within genome segments can have considerable longevity (from 3 to 7 decades, see Table 2), but few genome constellations from prior to 1983 were still present in the 1990s, suggesting that reassortment is a common event in Influenza C evolution.  A closer analysis of the older strains reveals that the previous terminology for the constellations may lead to confusion.  For instance, constellation 3 defined here is equivalent to the “AI81-related lineage” [4].  However, strain C/Aichi/1/81 itself must be regarded as an outlier for genome segments 1, 3 and 7.  Likewise constellation 2 defined here is equivalent to the “MS80-related lineage” [4].  However, strain C/Mississippi/80 itself has a C/Greece/79-lineage sequence in fragments 1 and 2 and a C/Aomori/74-lineage sequence in segment 7.  Again, constellation 1b defined here is equivalent to the “YA2681-related lineage” [4].  However, strain C/Yamagata/26/81 itself has a C/Sapporo/71-lineage sequence in fragment 3 and a C/Shizuoka/79-lineage sequence in segment 5.  In fact, strain C/Yamagata/26/81 is closer to constellation 6 (represented by C/Miyagi/3/91) having six out of seven segments in the same lineage, than it is to constellation 1b.     

**Figure:**
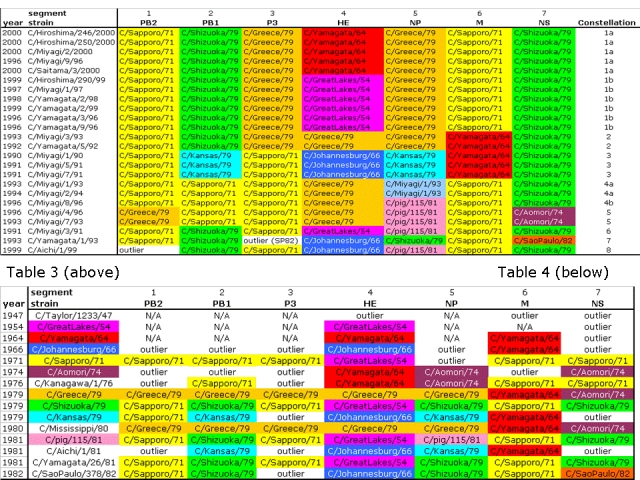
Table 3: 24 of the most recent completely sequenced strains, each with its lineage indicated (see Table 2) for all 7 segments.  Strains C/Aichi/1/99 and C/Yamagata/93 are outliers for segments 1 and 3 respectively, although the latter has a segment 3 sequence identical to strain C/Sao Paulo/82. 

Table 4:  15 reference strains, each with its lineage indicated (see Table 2) for all 7 segments.  In the “strain” column, strain names are coloured if they are the exemplary strains of a lineage in one or more segments.  N/A: sequence not available.

### Most Recent Common Ancestors of Each Segment 

Bayesian analysis of the substitution rate in each of the seven genome segments permits an estimation of the t-MRCA for each (Table 5).  The estimate of a rate of 4.87 x 10^-4^ (95% HPD: 4.12 x 10^-4^ to 5.66 x 10^-4^) substitutions per site per year for genome segment 4 is highly congruent with previous estimates of 4.9 x 10^-4^ substitutions/site/year in the HE gene [17].  Genome segment 2 is the fastest evolving segment, with a mean substitution rate of 6.77 x 10^-4^ per site per year (95% HPD: 4.78 x 10^-4^ to 8.89 x 10^-4^).  This is nearly double that of the slowest evolving segment 7.  However, there is considerable overlap in the 95% confidence intervals (Figure 2).  It is also noticeable that segment 3 has a wider range of 95% HPD than the others, possibly on account of its smaller sample size of only 39 sequences.  Where the mean substitution rate for one segment falls outside of the 95% HPD of another segment, a significant difference in rates may be inferred at the 5% significance level.  Using this method, segment 2 has a significantly higher substitution rate than segments 4, 1, 6 and 7, but not segments 5 and 3 (Table 5).  At the other end of the scale, segment 7 has a significantly lower substitution rate than segments 2, 5 and 4. 

**Figure:**
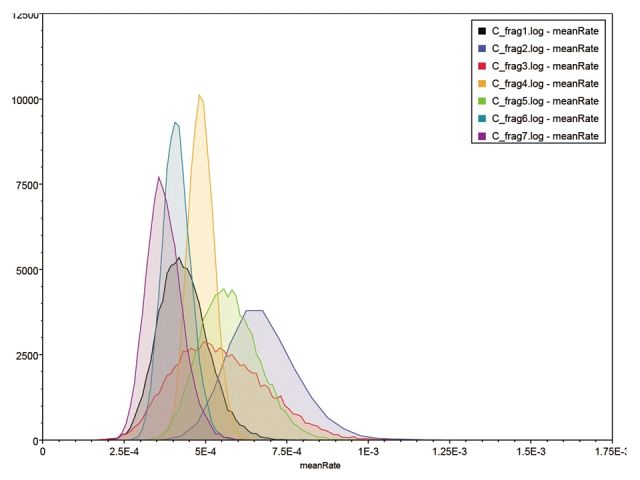
Figure 2:  Distribution of the substitution rates for the 7 genome fragments calculated over 10^8 ^iterations in BEAST and analysed using Tracer.  The y-axis gives the number of samples with each mean smoothed into 10 bins to generate a distribution curve.

**Figure:**
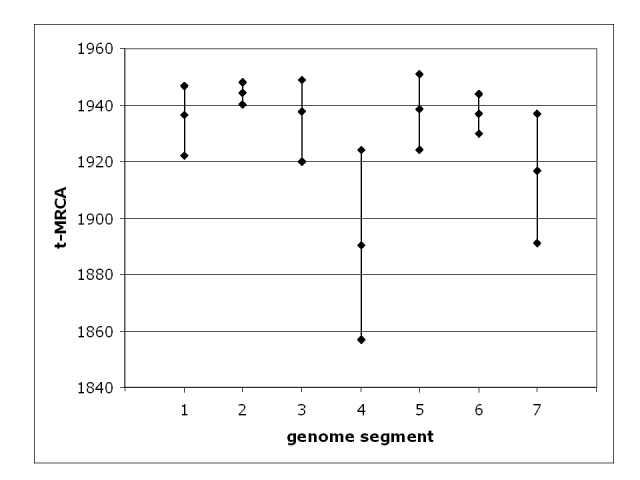
Figure 3: Probability distributions of t-MRCA values, expressed as calendar years (AD), for each of the seven genome segments.  The middle dot on each line is the mean and the upper and lower dots the 95% highest posterior density (HPD) limits.

The mean t-MRCAs of the individual segments range from the year 1890 for segment 4 to 1944 for segment 2 (Figure 3).  Most of the t-MRCAs are concentrated with means in the late 1930s or early 1940s, with 95% confidence limits from the 1920s to the late 1940s.  The two exceptions are segment 4 which has a mean t-MRCA in the 19^th^ century and segment 7 which is intermediate between segment 4 and the others.  Segment 4 is the most variable (Pi), with an average of 0.042 substitutions per site, more than twice the number of the least variable, segment 1 (Table 5).  The correlation coefficient between variability and t-MRCA is 0.74, significant as assessed by t-test at *p*<0.05, suggesting that sequence diversity accumulates with age.  As performed above for the substitution rates, where the mean t-MRCA for one segment falls outside of the 95% HPD of another segment, a significant difference may be inferred at the 5% significance level.  Using this method, segment 4 has a significantly older MRCA than all the others, although this is borderline in the comparison between segment 4 and segment 7 (Table 5).  Segment 7 has a significantly older MRCA than segments 2, 5, 3, 1 and 6.  The t-MRCA mean values may therefore be partitioned into two groups, a set of five segments and the pair of segment 7 and segment 4.  Even without statistical analysis, this is intuitively obvious from Figure 3.  

**Figure:**
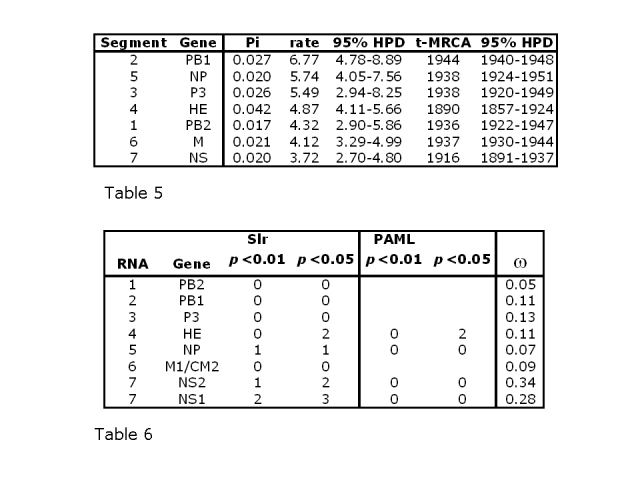
Table 5: Nucleotide substitution rates in substitutions x 10^-4^ per site per year and time of most recent common ancestor (t-MRCA) with their respective 95% highest posterior densities (HPD).  Segments are arranged in descending order of substitution rate.  Pi: average number of substitutions per site in the alignment.  The correlation coefficient between variability and t-MRCA is 0.74 (significant at *p*<0.05), suggesting that sequence diversity accumulates with age.

Table 6: Positive selection analysed using Slr and PAML, showing numbers of sites predicted to be under positive selection at 5% and 1% significance levels.  PAML was only run where Slr initially gave a positive score. ω: average non-synonymous to synonymous substitution rate ratio (dN/dS) for each alignment (omega).

### Natural Selection 

Table 6 shows the number of sites where positive selection was detected using Slr, and if a positive result was obtained, checked with PAML.  Using Slr, the HE, NP, NS2 and NS1 cDNA alignments all had at least one candidate site for positive selection at the 5% significance level, with NP, NS1 and NS2 also having sites significant at the 1% level.  However, on further examination with PAML, only HE was positive, and only at the 5% level.  The candidate positively selected sites are at positions 172 and 194 of HE. 

Average ω is never more than 0.35 for any of the cDNA alignments and in all the genes except NS1 and NS2 is less than 0.14, indicating that moderate constraint is the prevailing mode.  The substitution rates in the three codon positions for NS2, the gene with the highest average ω, are shown in Figure 4.  The third codon position has a substitution rate slightly greater than double that in the first and second positions.  The equivalent plots for the other 7 genes are given as Supplementary Figure 7.  In all cases, the substitution rate is higher in the third position than in the other two positions.

**Figure:**
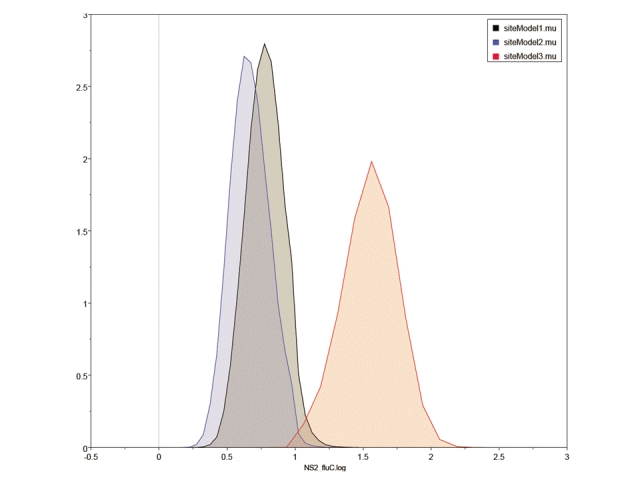
Figure 4:  Distribution of the substitution rates for the NS2 gene in the three codon positions, calculated over 10^8^ iterations in BEAST and analysed using Tracer.  The y-axis gives the relative number of samples with each mean smoothed into 10 bins to generate a distribution curve.

PAML also allows a statistical test between different models of selection [26] [35].  For all cDNA alignments, the following models were simulated: M0 (single ω), M1 (2 categories: ω < 1 and ω = 1), M2 (3 categories: ω < 1, ω = 1 and ω >1), M3 (3 categories, no constraints), M7 (10 categories, beta distributed from ω = 0 to ω = 1), M8 (M7 plus an additional category: ω > 1), M8a (M7 plus an additional category: ω = 1).  The following pairs were tested: M0 vs. M3 (test for uniform versus multiple categories of ω), M1 vs. M2 (test of selection versus neutrality with discrete categories of ω), M7 vs. M8 (test of selection versus neutrality with beta distribution of ω), M8 vs. M8a (test for strong vs. weak selection).  NS1, NS2 and HE gave statistically significant results (*p*<0.003 in all cases) for test M0 vs. M3 only, indicating a significant possibility of variable categories of ω along these alignments.  However, none of the other tests gave statistically significant differences in probability for any of the cDNA alignments.  

The X-ray diffraction structure of the HE protein from the C/Johannesburg/1/66 strain has been solved [36] (PDB reference 1FLC).  Both candidate positively selected residues, at positions 172 and 194, are in the receptor domain of the protein (Figure 5), and 172 is part of the receptor binding site [36].      

**Figure:**
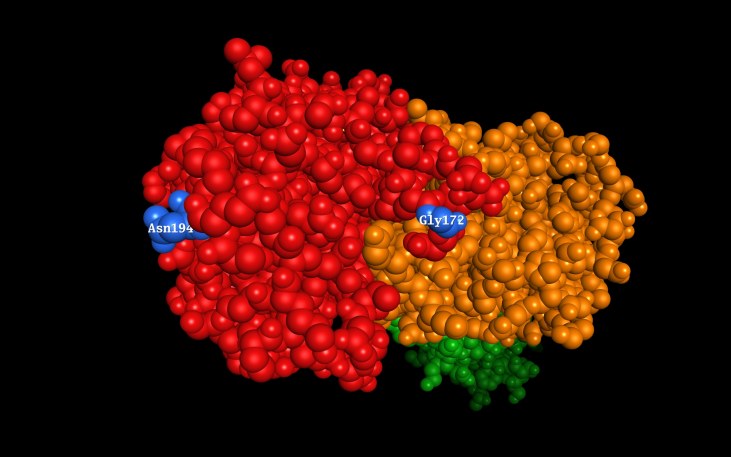
Figure 5:  Solved structure of HE (PDB 1FLC), viewed from receptor end with slight tilt to left, with candidate positively selected positions 172 and 194 indicated (blue).  Red: receptor-binding domain; orange: receptor-destroying (esterase) domain; green: membrane fusion domain (stem).

## Discussion 

Each of the seven genome segments of Influenza C may be considered, by way of null hypothesis, as evolving independently.  In the absence of recombination, under a neutral model, each would accumulate substitutions at its own rate, which again may be initially assumed to be roughly equal across all segments.  Stochastic birth and death of lineages would occur, and older clades would be the most diverse in terms of number of strains and their average sequence diversity.  This is the case for Influenza C, where the correlation coefficient between size of an individual lineage (defined here as a high confidence clade, see Figure 1) within a segment and the t-MRCA of that lineage is 0.53 (significant at *p*<0.0001, Table 2), and that between total segment sequence variability (Pi) and the segment’s t-MRCA is 0.74 (significant at *p*<0.05, Table 5).  

Slightly less than a twofold difference is found in mean nucleotide substitution rates across the different genome segments of Influenza C.  The 95% HPDs of the substitution rates also show considerable overlap, indeed the lower tail of the fastest evolving segment touches the upper tail of the slowest (Figure 2 and Table 5).  Nevertheless, some statistically significant differences in substitution rate may be demonstrated between the fastest evolving segment 2 and four of the other segments (4, 1, 6 and 7).  Overall, the substitution rates in Influenza C are of a similar order of magnitude to those described for Influenza B but an order of magnitude slower than those of Influenza A [37].  This reflects the fact that both B and C are viruses that are normally exclusive to humans and likely to be well adapted to their hosts, whereas Influenza A has its natural reservoir in aquatic wildfowl and, even after novel pandemic influenza subtypes have settled into seasonal occurrence, is always in a more acute struggle with the human immune system.  In all seven genome segments, the substitution rate is lower in non-synonymous positions, suggesting that selective constraint is operating.  The overall ratio (ω) of non-synonymous to synonymous substitution rates in all seven genome segments is less than 0.35 and in six cases is less than 0.15 (Table 6).  PB2, NP and M1-CM2 appear to be the most constrained proteins with NS2 the most relaxed.  Additionally, statistical tests for positive selection give only weak results (Table 6) and even these must be open to doubt given the inability to ensure that strains were not cultured in embryonated hens eggs [27].  Nevertheless, the candidate positively selected residues (Figure 5) do not belong to N-glycosylation sites, previously shown to be targets for selection in egg culture in Influenza B [27].  Furthermore, both are in the receptor-binding domain of the HE protein and one is at the receptor-binding site.  It therefore remains possible that positive selection for host immune system evasion operates in Influenza C.  

Genome segments 4 and 7, encoding the the haemagglutinin-esterase (HE) protein and non-structural (NS) proteins respectively, have statistically significantly earlier t-MRCAs than the other five segments (Table 5).  The substitution rate of segment 4 is in the middle of the segment-specific range (Table 5), as is its ω value (Table 6).  Segment 4 does not therefore appear to be under any exaggerated constraint compared to the other segments and segment 7 is both the slowest evolving segment (Table 5) and under the weakest selective constraint as judged by its ω value (Table 6).  Positive selection analysis must be interpreted with caution, inhibited as it is by the caveats concerning artefactual signals produced by egg-culture selection [27].  However, both segment 4 and segment 7 have candidate positively selected residues as judged by Slr [25] although only segment 4 is positive as judged by PAML [26].   The possibility is therefore raised that some other mechanism other than direct selection on HE is needed to explain its coalescent depth relative to the other segments.  One possibility is that HE is less sensitive to the consequences of reassortment, which is common (Table 3 and Table 4). 

Reassortment has been a major feature of the evolution of Influenza C.  The nine genome constellations identifiable between 1990 and 2000 (Table 3) have only two representatives visible in the selection of strains taken from 1947 to 1982.  Lineages within segments, which may have t-MRCAs dating back to the 1940s (Table 4) are apparently continually reassorted through constellations that endure for little more than a decade or so at most.  The hypothesis could be tested by complete genome sequencing of more recent strains.  Only segment 4 has been sequenced in any strain later than 2001, and these newer sequences have revealed the continued existence of the C/Yamagata/64 and C/Johannesburg/66 lineages, having mean t-MRCAs of 1950 and 1941 respectively. 

Further analysis of more recent strains of Influenza C is justified not merely because of the economic burden of the virus, but also because its frequent reassortment provides an interesting natural laboratory for the evolutionary consequences of that process.  Insights from Influenza C may be of value in the study of its two more devastating relatives.    

## Acknowledgements

The author thanks colleagues at the MRC-University of Glasgow Centre for Virus Research for many stimulating discussions, in particular Prof. Duncan McGeoch and Dr Andrew Davison for providing the space and time in which the project could be done and Prof. Bill Carman for first suggesting flu as a topic.  Prof. Dan Haydon, Dr Barbara Mable and their colleagues were also especially generous with their time and expertise.

## Funding Information

The UK Medical Research Council.

## Competing Interests

The author declares he has no competing interests.

## Supplementary Figures

**Figure:**
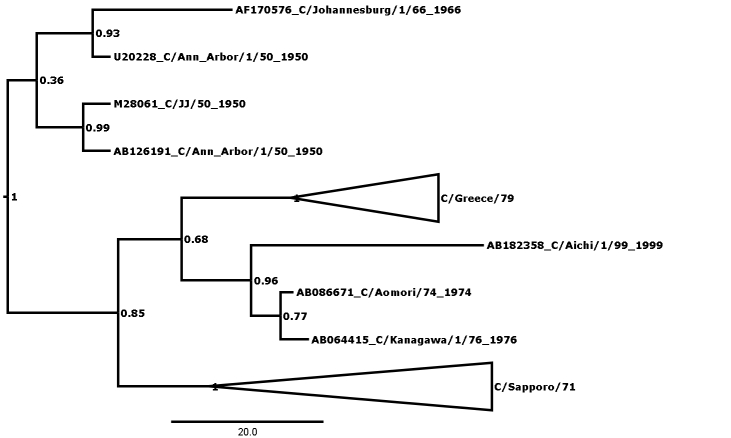
Supplementary Figure 1: Phylogenetic tree for segment 1, encoding basic polymerase protein 2 (PB2).  Bayesian posterior confidence values for the clades are given at each node.  Lineages (see Table 2) are collapsed into triangles.  The 7 outlier strains (see Table 1) are named in full.  The scale is in years.

**Figure:**
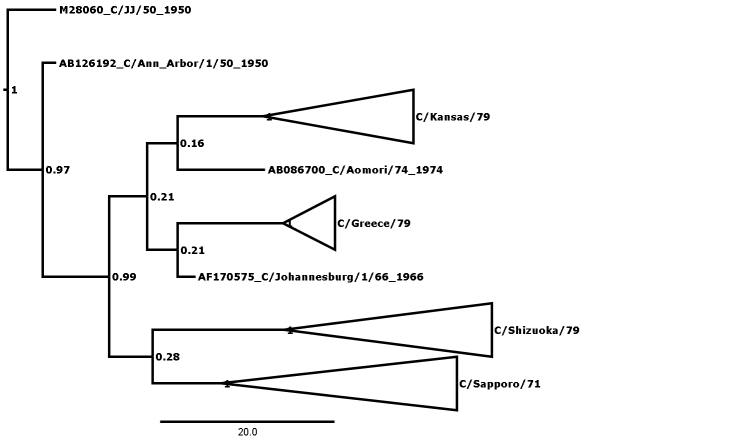
Supplementary Figure 2: Phylogenetic tree for segment 2, encoding basic polymerase protein 1 (PB1).  Bayesian posterior confidence values for the clades are given at each node.  Lineages (see Table 2) are collapsed into triangles.  The 4 outlier strains (see Table 1) are named in full.  The scale is in years.

**Figure:**
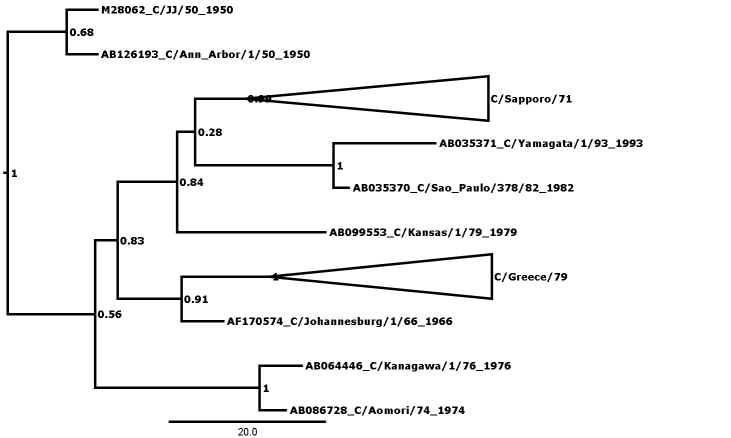
Supplementary figure 3: Phylogenetic tree for segment 3, encoding polymerase protein 3 (P3).  Bayesian posterior confidence values for the clades are given at each node.  Lineages (see Table 2) are collapsed into triangles.  The 8 outlier strains (see Table 1) are named in full.  The scale is in years.

**Figure:**
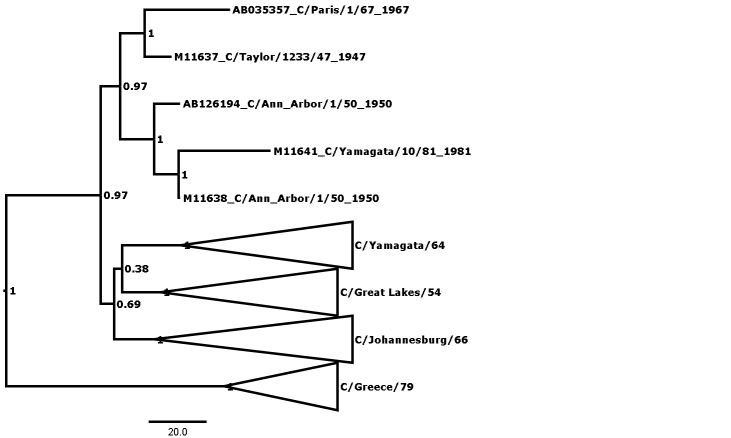
Supplementary Figure 4:  Phylogenetic tree for segment 4, encoding the haemagglutinin-esterase protein (HE).  Bayesian posterior confidence values for the clades are given at each node.  Lineages (see Table 2) are collapsed into triangles.  The 5 outlier strains (see Table 1) are named in full.  The scale is in years.

**Figure:**
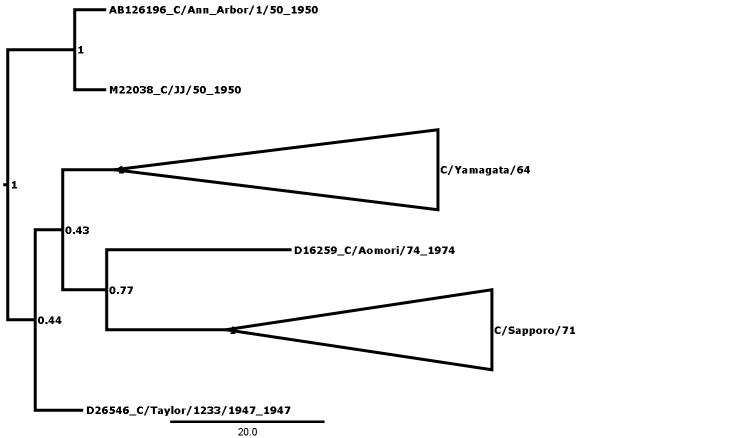
Supplementary Figure 5: Phylogenetic tree for segment 6, encoding the matrix protein (M).  Bayesian posterior confidence values for the clades are given at each node.  Lineages (see Table 2) are collapsed into triangles.  The 4 outlier strains (see Table 1) are named in full.  The scale is in years.

**Figure:**
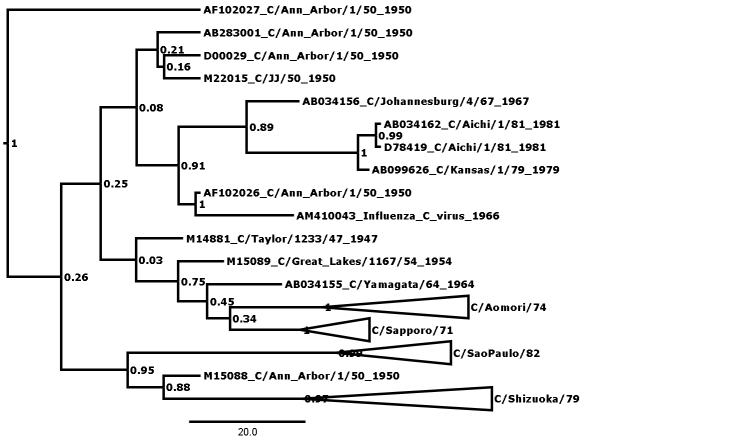
Supplementary figure 6: Phylogenetic tree for segment 7, encoding the non-structural proteins (NS1 & NS2).  Bayesian posterior confidence values for the clades are given at each node.  Lineages (see Table 2) are collapsed into triangles.  The 14 outlier strains (see Table 1) are named in full.  The scale is in years.

**Figure:**
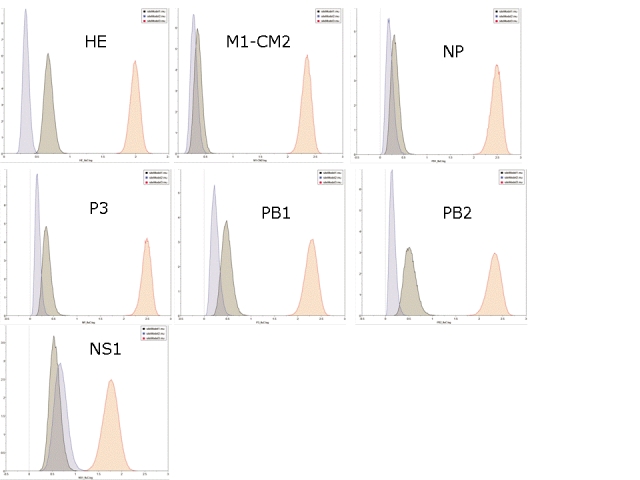
Supplementary Figure 7: Distribution of the substitution rates for all genes except NS2 (see Figure 4) in the 3 codon positions, calculated over 10^8^ iterations in BEAST and analysed using Tracer.  The y-axis gives the relative number of samples with each mean smoothed into 10 bins to generate a distribution curve.
